# Correction Notice: Labeling Aversive Memory Trace in Mouse Using a Doxycycline-inducible Expression System

**DOI:** 10.21769/BioProtoc.3310

**Published:** 2019-07-05

**Authors:** Erin E. Koffman, Jianyang Du

**Affiliations:** Department of Biological Sciences, University of Toledo, Toledo, OH, USA

I published the article (https://bio-protocol.org/e2578) in 2017. In this paper, I was the corresponding authors and my name should be in the second position (senior author). I mistakenly put my name first. I thus would like to switch the order of the authorship with my student, Ms. Erin Elizabeth Koffman. Please see the new order below. The new Authorship: Erin Elizabeth Koffman and Jianyang Du*.

In addition, this work and the continued works were partially supported by NIH 5R01MH113986. We thus amend the acknowledgment part as follow:

We thank Thomas Moninger, Theresa Mayhew, and Sarah Horgen for assistance. We thank Drs. Susumu Tonegawa, Xu Liu, and Steve Ramirez for providing the TRE-mCherry plasmid and their previous works (Liu et al., 2012; Ramirez et al., 2013). JD is supported by the American Heart Association (15SDG25700054), the University of Toledo start-up fund and **NIH 5R01MH113986**.

## 
References



Koffman, E. E. and Du, J. (2017).  Labeling Aversive Memory Trace in Mouse Using a Doxycycline-inducible Expression System. 
*Bio-protocol* 7(20): e2578.


